# Interacting Effect of Catechol-*O*-Methyltransferase (*COMT*) and Monoamine Oxidase A (*MAOA*) Gene Polymorphisms, and Stressful Life Events on Aggressive Behavior in Chinese Male Adolescents

**DOI:** 10.3389/fpsyg.2018.01079

**Published:** 2018-07-03

**Authors:** Meiping Wang, Hailei Li, Kirby Deater-Deckard, Wenxin Zhang

**Affiliations:** ^1^Department of Psychology, Shandong Normal University, Jinan, China; ^2^Department of Business, Shandong Normal University, Jinan, China; ^3^Department of Psychological and Brain Sciences, University of Massachusetts, Amherst, MA, United States

**Keywords:** MAOA, *COMT*, stressful life events, aggressive behavior, gene × gene × environment interaction

## Abstract

Numerous studies have demonstrated that both catechol-*O*-methyltransferase (*COMT*) gene and monoamine oxidase A (*MAOA*) gene have been involved in aggressive behavior, as have stressful life events (SLEs). However, most of available evidence was based upon single gene or single gene–environment design, which is limited in accounting for the variance of aggressive behavior, a complex phenotype. This study examined the possible gene × gene × environment interactions between SLE (interpersonal problems and academic pressure) and two genetic polymorphisms (*MAOA* T941G and *COMT* Ala22/72Ser) correlated with aggressive behaviors in a sample of 658 Chinese male adolescents. Mothers and teachers reported on adolescents’ aggressive behavior using Achenbach’s Child Behavior Checklist and Teacher Report Form, respectively. Adolescents completed Self-Rating Life Events Checklist. Saliva samples were collected for DNA analysis. The results revealed no main effects of *MAOA* T941G and *COMT* Ala22/72Ser polymorphisms on male adolescents’ aggressive behaviors. However, a two-way interactive effect of interpersonal problems and *MAOA* T941G genotype on teacher-reported aggressive behavior was observed: adolescents with lower activity of *MAOA* T allele, but not those with *MAOA* G allele, exhibited greater aggressive behavior with an increase in interpersonal problems. A three-way interaction among *COMT* Ala22/72Ser and *MAOA* T941G polymorphisms, and SLE in the academic pressure on aggressive behavior was also identified. Among adolescents with lower activity of *COMT* GT/TT genotype and *MAOA* T allele, the higher level of academic pressure was significantly linked with an amplification of aggressive behavior, whereas this association didn’t exist among those with other genotypes. The present study presents the first evidence of *COMT* × *MAOA* × SLE interaction effect on male adolescents’ aggressive behavior, highlights the importance of considering distinct domains of stressful events and information bias when examining the effect of *MAOA* and *COMT* on aggressive behavior, and thereby contributes to *MAOA* gene-aggression and *COMT* gene-aggression literature.

## Introduction

Aggressive behavior is a common externalized problem behavior which exerts a short-term and long-term negative effects on the development of adolescents (Ritakallio et al., 2005; Card et al., 2008). Therefore, it would be both interesting and necessary to identify the the underlying mechanism that contributes to the development of adolescent aggressive behavior. During the past decade, there have been a number of studies have exmined the risk factors for aggressive behavior in adolescence. Previous research has demonstrated that individual differences in such behaviors have important genetic as well as environmental underpinnings (Caspi et al., 2002; Brendgen et al., 2006; Craig and Halton, 2009; McDermott et al., 2009; Johansson et al., 2012; Zhang Y. et al., 2017), and a large body of the available evidence indicated that catecholaminergic system genes may interact with a variety of environmental exposure in predicting aggressive behavior (Perroud et al., 2010; Hygen et al., 2015; Zhang H. et al., 2017).

Among the catecholaminergic system genes, catechol-*O*-methyltransferase (*COMT*) has attracted most attentions. The human *COMT* gene is located on chromosome 22q 11.2 and encodes COMT, the major metabolic enzyme for catecholamines (adrenalin, noradrenaline, and dopamine). In most studies, the *COMT* gene polymorphism of particular interest was referred to as the Val158Met (rs4680) locus, a common polymorphism within the coding sequence of *COMT* (Montirosso et al., 2016; Sheikh et al., 2017; Zhang H. et al., 2017; Minassian et al., 2018). However, it should be noted that among Asians, including Chinese populations from which the current study’s sample was drawn, a second common functional single nucleotide polymorphism at which the variant between a G and T causes an alanine -to-serine (Ala/Ser) substitution, also exists in exon 4. This Ala/Ser variation was associated with a more dramatic reduction in COMT enzyme activity than the most studied Val/Met polymorphism, although *COMT* Ala22/72Ser polymorphism (rs6267) is not found in Caucasian or African American populations, who all (as far as is known) carry the alanine allele at this locus (Lee et al., 2005; Hong et al., 2008; Wang, 2009). A few of studies have found that a serine substitution at rs6267 was significantly associated with increased aggressive behavior (Hong et al., 2008; Wang and Zhang, 2010), however, there seems to be a long way to go given less research on gene–environment interaction has targeted this variant by far.

Monoamine oxidase A, strong candidate catecholaminergic system gene to be associated with aggressive behavior, is an X-linked gene, located on the X chromosome at Xp11.23-11.4. It encodes the mitochondrial enzyme *MAOA*, which plays a key role in the degradation of monoamines, including norepinephrine (NE), dopamine (DA), and serotonin (5-HT). *MAOA* knockout mice have been shown to exhibit elevated aggressive behavior (Cases et al., 1995), but results from studies of the association between *MAOA* and aggressive behavior in humans were still inconsistent. A broader literature has shown that the low-activity *MAOA* genotype is associated with more aggressive behavior (Caspi et al., 2002; Hart and Marmorstein, 2009; McDermott et al., 2009; Hohmann et al., 2016), while some other work has come to the opposite conclusion – that the high-activity *MAOA* genotype is associated with more aggressive behavior (Manuck et al., 2000; Beitchman et al., 2004; Kinnally et al., 2009; Mcgrath et al., 2012). Still other studies have indicated that *MAOA* gene polymorphisms are not associated with aggressive behavior (Fresan et al., 2007).

One possible explanation for the mixed results may be that the links between *MAOA* variants with aggressive behavior are heterogeneous and moderated by other genetic variants and non-genetic factors (i.e., environmental variables). A latest stuy (Zhang Y. et al., 2017) found a significant three-way interaction between *5-HTTLPR* (serotonin transporter gene linked polymorphism), *MAOA-*VNTR (upstream variable mumber tandem repeat) and childhood sexual abuse on aggressive behavior, such that the *5-HTTLPR* by sexual abuse interaction was limited to adolescents with the high-expression *MAOA-*VNTR. These results implicated the necessarity to simultanenously consider the impact of other genetic factors and environmental influences when examining a single gene “effect” for aggressive behavior.

Catechol-*O*-methyltransferase and MAOA, which are the major enzymes involved in the degradation of catecholamines, may jointly regulate catecholamine activities in the brain that are involved in aggressive behavior (Gogos et al., 1998; Volavka, 2002). Previous studies showed that the low-activity *COMT* genotype in combination with low-activity *MAOA* variant was linked with higher adrenocorticotrophin hormone (ACTH) responses (Jabbi et al., 2007) and cortisol levels (Bouma et al., 2012). Therefore, *COMT* gene might significantly moderate the interaction between *MAOA* gene and environment on adolescent aggressive behavior. However, to our knowledge, there are no published studies examining *MAOA* ×*COMT* × Environment interactions for aggressive behavior.

In addition, the majority of extant studies (Manuck et al., 2000; Caspi et al., 2002; Beitchman et al., 2004; Kinnally et al., 2009; Mcgrath et al., 2012; Hohmann et al., 2016) on *MAOA* gene have focused on the effects of a variable number tandem repeat (VNTR) polymorphism of 30-bp located in the upstream promoter region (*MAOA*-VNTR), which is associated with differences in MAOA activity. Another polymorphism, T941G (rs6323) is a common functional polymorphism in exon 8 of the *MAOA* gene; the TG or GG genotype codes for the higher activity form of the enzyme. The association between the *MAOA* T941G polymorphsim and behaviors such as borderline personality disorder (Ni et al., 2007), placebo responses of patients with major depression (Leuchter et al., 2009), and violent behaviors among prisoners (Liu et al., 2009), have been reported. However, like *COMT* Ala22/72Ser, relatively little is known regarding the association between *MAOA* T941G polymorphsim and adolescents’ aggressive behaviors. In the current study we employed these two gene polymorphisms to examine the possible gene × gene × environment interactions.

With respect to “candidate” environment factors, we have focused on stressful life events (SLEs). According to the frustration-aggression hypothesis, which holds that any form of negative affect or distress is possible to increase the likelihood of aggression, SLE could thus produce aggressive behavior because they create negative affect. Empirical studies (Guerra et al., 1995; Zhou et al., 2008; Hygen et al., 2015) also have showed that SLEs are associated with the etiology of aggression and externalizing problems. The results of these prior studies are informative, but these studies were mainly guided by gene–environment (G × E) interaction models that used total or overall SLE aggregated across multiple domains of the environment (e.g., peers, parents, school environment, home environment). This is potentially problematic, because recent evidence indicated that the role SLE played in gene–environment mechanisms may be domain specific. For instance, Vaske et al. (2009) found that the *DRD2* TaqIA polymorphism interacted with witnessed violence, but not weak attachment to others to predict higher levels of depressive symptoms. Therefore, the potential interactions among SLE and the *COMT* and *MAOA* polymorphisms may vary across distinct domains of stressful events, however, to our knowledge, this assumption still remains to be studied. The stressors included in the present study are limited to interpersonal problems and academic pressure, because previous research has indicated that in China both of the stressors are two SLEs that adolescents are frequently exposed to Liu et al. (1997) and Guo et al. (2009), and furthermore may cause even greater problems for Chinese adolescents (Zhang et al., 2013).

In summary, the bulk of existing studies on the association between *MAOA* or *COMT* gene polymorphisms and aggressive behavior were investigated separately. Gene-gene-environment (G × G × E) interactions were neglected in most of the studies. The present study was designed to examine the association among two genetic polymorphisms within the catecholaminergic systems (*COMT* Ala22/72Ser and *MAOA* T941G) and SLEs (interpersonal problems and academic pressure) with aggressive behaviors in a general population sample of Chinese male adolescents.

## Materials and Methods

### Participants

A total of 1294 adolescents (658 boys and 636 girls, average age 15.26 ± 0.29 years) and their mothers (average age 42.16 ± 2.54 years) were drawn from 97 classes of 11 junior high schools in Jinan, the capital city of Shandong Province in eastern China. Ninety-seven percentage of the participants were of Chinese Han ethnicity. Since *MAOA* T941G is X-linked and unequivocal information about X-inaction at this locus is not available, we limited our analyses to males (658). There were no statistically significant differences in the demographic variables between these 658 boys and the unselected sub-sample from the original participants.

### Procedure

Prior to data collection, we gained an approval for the questionnaire and saliva sampling from ethics committee of Shandong Normal University. All data collection was carried out by a group of trained researchers. Written informed assent from adolescents and consent from their mothers were also obtained prior to the start of data collection. At the appointed time with the head teacher who was in charge of a class, we administered in group to adolescents a self-reported questionnaire of SLEs during class hours. Mothers were asked to complete the CBCL (The Child Behavior Checklist, Achenbach, 1991a), which was taken home by adolescents and returned to head teacher on the following day. Head teachers rated adolescents’ aggressive behaviors using TRF (Achenbach, 1991b) at their offices. About 2 weeks later, adolescents were asked to provide their saliva samples for DNA analysis under the detailed instructions of trained investigators.

### Behavioral Measurements

#### Aggressive Behavior

Data was collected by Achenbach’s Child Behavior Checklist (CBCL/6-18; Achenbach, 1991a), and Teacher Report Form (TRF/5-18; Achenbach, 1991b). Mothers assessed adolescents’ aggressive behavior by 20-item CBCL Ratings for each item ranged from 0 ‘not true,’ and 1 ‘somewhat or sometimes true’ to 2 ‘very true or often true.’ The TRF includes 25 items and teachers were asked to rate adolescent behavior as 0, 1, or 2, like the rating of the CBCL, based on a 2-month period. Cronbach’s alpha values for the two subscales were 0.81 and 0.88, respectively. Correlation between mother and teacher reports was 0.29 (*p* < 0.001), and therefore these two sources of data, but not the aggregate score was analyzed respectively.

#### Stressful Life Events

Interpersonal problems (item = 5, e.g., ‘Misunderstood/rejected by others’ and ‘Break up with close friends.’) and academic pressure (item = 4, e.g., ‘Homework overload,’ and ‘Entrance examination pressure.’) were measured with items from Adolescent Self-Rating Life Events Checklist (ASLEC), which was designed to measure exposure to SLEs and has been tested in studies with Chinese Adolescents (e.g., Liu et al., 1997; Chen et al., 2012). The adolescents were required to indicate whether each event had occurred in the past 12 months or not using a scale ranging from 1 (no impact) to 5 (extremely serious impact). Item scores were averaged to compute each subscale scores. Cronbach’s alpha values for the two subscales were 0.77 (interpersonal) and 0.76 (academic), respectively.

#### Genotyping

DNA was isolated from saliva sample collected with Oragene collection kits (DNA Genotek, Ottawa, ON, Canada) using the Klear-gene DNA extraction method. The single nucleotide polymorphism (SNP) genotyping was performed using the MassARRAY system (Sequenom, United States) by means of matrix assisted laser desorption ionization-time of flight mass spectrometry method (MALDI-TOF) according to the manufacturer’s instructions. The *COMT* rs6267 polymorphism was amplified using forward primer ACGTTGGATGTAGGTGTCAATGGCCTCCAG and reverse primer ACGTTGGATGTCATGGGTGACACCAAGGAG. The primer sequence for *MAOA* T941G polymorphism was as follows: forward ACGTTGGATGTGCACTTAAATGACAGTCCC and reverse ACGTTACGTTGGATGGATTCACTTCAGACCAGAGC. All genotyping was done blind to all the other variables (e.g., stressful life events and aggressive behavior).

### Data Analyses

Given there were only a small number of TT genotypes, and the TT genotype codes for the similar activity form of the enzyme as GT genotype (Lee et al., 2005), these two genotypes were pooled for the purpose of analyses, which is common in previous studies (e.g., Windle and Mrug, 2015; Zhang H. et al., 2017). Bivariate Pearson correlations were conducted to assess the associations between observed variables in the present study. A set of hierarchical linear regression analyses were employed to examine the effects of *COMT* Ala22/72Ser and *MAOA* T941G genotype, and SLEs in academic or interpersonal domains on male adolescents’ aggressive behaviors. In each analysis, main effect terms were entered on the first step, the two-way interaction terms on the second, and the three-way interaction term on the third. *p*-Values were adjusted to control the false discovery rate using Benjamini and Hochberg (1995) procedure.

## Results

Since *MAOA* T941G polymorphism is X-linked and males had only one allele, the participants were assigned to G (*n* = 393; 60%) or T (*n* = 265; 40%) group (see **Table 1**). The genotype frequencies of the *COMT* rs6267 polymorphism were GG: 83%, GT: 16%, TT: 1%, which were in Hardy–Weinberg equilibrium (χ^2^ = 0.15, *p* = 0.70). Chi-square test indicated that there was no significant association between *MAOA* T941G and *COMT* Ala22/72Ser genotype [χ^2^(1) = 0.93, *p* = 0.39]. Descriptive statistics and intercorrelations of the variables included in this study are presented in **Table 2**. There were no significant correlations between *COMT* Ala22/72Ser polymorphism, *MAOA* T941G polymorphism and each measure of SLEs, suggesting no gene–environment correlations. Significant positive associations were o found between interpersonal and academic life events and mother-reported aggressive behavior, and between interpersonal problems and teacher-reported aggressive behavior, however, academic pressure was not significantly related to aggressive behavior by teacher reports.

**Table 1:** The distribution of *COMT* Ala22/72Ser and *MAOA* T941G polymorphisms.

Variable		*MAOA* T941G		Total
		G	T	
*COMT* Ala22/72Ser	GT/TT	69	39	108
	GG	324	226	550
Total		393	265	658

**Table 2:** Bivariate correlations, means, and standard deviations for study variables.

Variable	1	2	3	4	5	6
(1) *COMT* (0 = GG; 1 = GT/TT)	1					
(2) *MAOA* (0 = G; 1 = T)	-0.04	1				
(3) Academic pressure	-0.05	-0.05	1			
(4) Interpersonal	-0.06	-0.02	0.58^∗∗∗^	1		
(5) Mother-reported aggressive behavior	0.00	0.08	0.17^∗∗∗^	0.24^∗∗∗^	1	
(6) Teacher-reported aggressive behavior	0.02	0.06	0.06	0.15^∗∗∗^	0.29^∗∗∗^	1
*M*	–	–	6.53	8.80	3.27	2.56
*SD*	–	–	2.78	2.91	3.24	2.33

*^∗∗∗^p < 0.01.*

The findings of hierarchical regression analyses are summarized in **Table 3**. Interpersonal problems could positively predict aggressive behavior of both mother and teacher reports, and academic pressure could positively predict mother-reported aggressive behavior. No main effects of *COMT* Ala22/72Ser genotype and *MAOA* T941G genotype on aggressive behavior were found, however, a two-way interaction between the interpersonal problems and *MAOA* T941G genotype in the association with teacher-reported aggressive behavior was observed. Follow-up simple slope analyses indicated that among *MAOA* T carries increased interpersonal problems were significantly associated with more aggressive behavior (β = 0.26, *t* = 4.34, *p* < 0.001), but this association was not found among *MAOA* G allele carries (β = 0.07, *t* = 1.28, *p* = 0.20). Although there was no significant two-way interaction between *COMT* Ala22/72Ser genotype and each measure of SLEs on aggressive behavior, a significant three-way interaction effect among *COMT* Ala22/72Ser genotype, *MAOA* T941G genotype, and the academic pressure was observed on mother-rated aggressive behavior.

**Table 3:** Hierarchical linear regression analyses: interaction among *MAOA* T941G and *COMT* Ala22/72Ser genotypes and stressful life events on aggressive behavior.

Variable	Δ*R*^2^	ΔF	β	*T*	*P*	p(i)
**Mother-reported aggressive behavior**						
**Interpersonal problems**						
* COMT*	6.70%	15.50^∗∗∗^	0.02	0.41	0.68	0.05
* MAOA*			0.08	2.09	0.04	0.02
Interpersonal			0.25	6.28	<0.001	0.00
* MAOA* ×*COMT*	0.40%	0.96	0.01	0.22	0.83	0.05
* COMT* × Interpersonal			0.07	1.70	0.09	0.03
* MAOA* × Interpersonal			0.02	0.46	0.65	0.05
* MAOA* ×*COMT* × Interpersonal	0.40%	2.61	0.06	1.62	0.11	0.03
**Academic pressure**						
* COMT*	3.50%	7.80^∗∗∗^	0.03	0.76	0.45	0.04
* MAOA*			0.08	2.17	0.03	0.01
Academic			0.17	4.21	<0.001	0.00
* MAOA* ×*COMT*	0.30%	0.67	0.02	0.53	0.60	0.05
* COMT* × Academic			0.08	2.02	0.04	0.02
* MAOA* × Academic			0.02	0.61	0.55	0.04
* MAOA* ×*COMT* × Academic	1.0%	6.63^∗∗^	0.11	2.58	0.01	0.01
**Teacher-reported aggressive behavior**						
**Interpersonal**						
* COMT*	2.6%	5.789^∗∗^	0.03	0.674	0.50	0.04
* MAOA*			0.07	1.79	0.07	0.03
Interpersonal			0.17	4.30	<0.001	0.00
* MAOA* × *COMT*	1.5%	3.474^∗^	0.03	0.64	0.52	0.04
* COMT* × Interpersonal			0.05	1.30	0.20	0.03
* MAOA* × Interpersonal			0.11	2.73	0.01	0.01
* MAOA* × *COMT* × Interpersonal	0.2%	1.633	0.05	1.28	0.20	0.03
**Academic**						
* COMT*	0.8%	1.76	0.04	0.90	0.04	0.02
* MAOA*			0.08	1.93	0.05	0.02
Academic			0.09	2.21	0.03	0.01
* MAOA* × *COMT*	0.7%	1.59	0.05	1.27	0.20	0.03
* COMT* × Academic			0.04	0.86	0.39	0.04
* MAOA* × Academic			0.08	2.08	0.04	0.02
* MAOA* × *COMT* × Academic	0.8%	5.30^∗^	0.10	2.30	0.02	0.01

*^∗^p < 0.05, ^∗∗^p < 0.01, ^∗∗∗^p < 0.001.*

**FIGURE 1:**
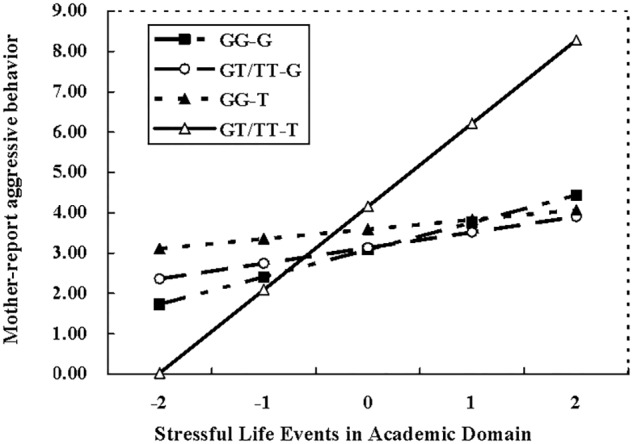
*MAOA* × *COMT* × SLE in the academic domain for mother-reported aggressive behavior. G-GG = *MAOA* G with *COMT* GG, G-GT/TT = *MAOA* G with *COMT* GT/TT, T-GG = *MAOA* T with *COMT* GG, T-GT/TT = *MAOA* T with *COMT* GT/TT.

To clarify this interaction, the effects of *MAOA* T941G genotype, academic pressure, and their interactions on aggressive behavior were examined among *COMT* GG carries and *COMT* GT/TT carries, respectively. For adolescents with *COMT* GG genotype, there was no significant interaction between *MAOA* T941G genotype and academic pressure (β = -0.68, *t* = -1.54, *p* = 0.32), however, such interactional effect was found for adolescents carrying *COMT* GT/TT allele (β = 0.24, *t* = 2.04, *p* < 0.05). Follow-up simple slope analyses indicated that the higher level of academic pressure was significantly linked with an increasing of aggressive behavior among adolescents with *COMT* GT/TT genotype and *MAOA* T allele (β = 0.43, *t* = 2.83, *p* < 0.01), whereas this association didn’t exist among those with *COMT* GT/TT genotype and *MAOA* G allele (β = 0.10, *t* = 0.83, *p* > 0.05) (**Figure 1**). In addition, the three-way interaction of *MAOA* ×*COMT* × Interpersonal problems did not significantly predict aggressive behavior of both mother and teacher reports.

Given the distributions of the SLEs and aggressive behavior were slightly positive skewed, we therefore took the square root of these variables—a common transformation method for the non-normal distribution of data—and reran a series of hierarchical regression analyses. The results indicated that the above mentioned main and interactional effects of the genes and SLEs on aggressive behavior remained stable even after the normalization of the distributions.

## Discussion

The purpose of this study was to explore the interaction effects of *COMT* Ala22/72Ser and *MAOA* T941G polymorphisms, two important catecholamine-related genes, and adolescents’ experience of SLE on aggressive behavior using a gene × gene × environment design in a general population sample of Chinese male adolescents. Neither *COMT* Ala22/72Ser nor *MAOA* T941G polymorphism was found to have a direct impact on aggressive behavior; however, the interactive effect of *MAOA* T941G polymorphism with interpersonal problems on teacher-rated aggressive behavior was uncovered. Moreover, *COMT* Ala22/72Ser polymorphism was discovered to play a moderating role on the interaction between *MAOA* T941G polymorphism and academic pressure in association with mother-reported aggressive behavior. These findings even remain robust after correction for multiple testing. To our knowledge, this is the first report of an interaction among *COMT* and *MAOA* gene and SLE predicting adolescents’ aggressive behaviors. Thus, the conclusion should be made with caution. In spite of this, our findings highlight the importance of examining the multi-genes by environment interaction on aggressive behavior and afford some explanations for the inconsistence of the findings from previous studies concerning the effects of *COMT* gene or *MAOA* gene on aggressive behavior. Our findings also lend further support for the theory that catecholamines may play an important role in adolescent aggressive behavior, and thereby contribute to *MAOA* gene-aggression and *COMT* gene-aggression literature.

In line with previous findings that a single gene polymorphism was not on its own related to aggressive behavior (e.g., Hygen et al., 2015; Hohmann et al., 2016; Tuvblad et al., 2016; Zhang Y. et al., 2017), our results didn’t show the main effect of *MAOA* T941G or *COMT* Ala22/72Ser polymorphism. However, *MAOA* T941G polymorphism was found to moderate the effect of interpersonal problems experienced by adolescents on aggressive behavior, specifically, adolescents with T allele were more likely to evince aggressive behavior when exposed to interpersonal problems. In contrast, among those with G allele, interpersonal problems could not predict aggressive behavior. An existing study demonstrated that adolescents carrying T allele of *MAOA* T941G gene exhibited more or less reactive aggression under conditions of low or high positive parenting (Zhang et al., 2016). Our findings seem to lend further support for the hypothesis that T allele of *MAOA* T941G gene might be the susceptible gene for aggressive behavior.

More importantly, the interaction between *MAOA* T allele and aggressive behavior under the stressful conditions depends on the genotype of *COMT* Ala22/72Ser, such that only among adolescents with GT heterozygote or TT homozygote of *COMT* gene, *MAOA* T allele carriers would display a higher inclination toward aggressive behavior when they were exposed to academic pressure. The underlying mechanism might be related to the catecholamine system. Variants in the *MAOA* and *COMT* genes play a critical role in regulating the activity of catecholamine including dopamine, noradrenaline and adrenaline, which were found to be involved in the etiology of aggressive behavior by a large number of studies (Miczek and Yoshimura, 1982; Miczek and Haney, 1994; Harrison et al., 1997; Patki et al., 2015). For *COMT* gene, the GT heterozygote or TT homozygote were associated with reduced COMT enzyme activity (Lee et al., 2005). For *MAOA* gene, the T allele encodes for lower activity of *MAOA* enzyme (Hotamisligi and Breakefiedl, 1991). Therefore, the *COMT* GT/TT genotype in combination with *MAOA* T allele was supposed to be linked with higher level of trans-synaptic catecholamines, mainly dopamine. There were evidences showing that individuals carrying the low-expressing *MAOA* gene and the low-activity *COMT* gene exhibited increased hypothalamo-pituitary-adrenal-axis response when exposed to a social stressor (Jabbi et al., 2007; Bouma et al., 2012). Another study also revealed that women with *MAOA* and *COMT* low-activity alleles scored higher for postpartum depression symptoms (Comasco et al., 2011). These findings might suggest that low-activity *MAOA* and *COMT* genes were more sensitive to environmental stimulus or life events given their elevated or supra-optimal catecholamine availability, and thereby adolescents with these kinds of genotypes exhibited higher levels of aggressive behavior. To be noted, this does not mean that adolescents with other genotypes were entirely non-susceptible to environmental effects but rather that different adolescents might respond to different levels of or types of environments. Whether adolescents will evince aggressive behaviors might depend on the combined action of genes and environmental factors on catecholamine activity in the brain. Further studies will be called for to gain insight into the mediating role of catecholamine availability between these two candidate gene polymorphisms and aggressive behavior.

We also found that for mother-reported aggressive behavior, interpersonal problems had positively predictive effect regardless of the *MAOA* or *COMT* genotype, even after the significant level correction; however, the effect of academic pressure varied across different genotype groups. For teacher reports, higher levels of interpersonal problems were also found to be linked with greater aggressive behavior, and this association was moderated by *MAOA* genotype, but academic pressure was not an important predictive factor of teacher-reported aggressive behavior. The explanations for the above differences might be as follows: firstly, as expected, these findings verified that the effect of SLEs on aggressive behavior would vary across distinct domains, which highlights the necessity to differentiate various subtypes of SLEs in future studies; secondly, compared to academic pressure, interpersonal problems played a stronger role in predicting adolescent aggressive behavior, therefore, improving adolescents’ interpersonal relationships might be an effective way to prevent aggressive behavior (Nie et al., 2012); thirdly, informant bias might be partially responsible for the differences in the main effects of SLEs in interpersonal and academic domains, and their interaction effects with *MAOA* and/or *COMT* genotype. Each informant usually reports aggressive behavior based on their own observations and their relationships with adolescents, which results in the levels of aggressive behaviors from mother reports and teacher reports are often distinct (Hudziak et al., 2003), just as shown in our study (correlation between mother reports and teacher reports was 0.29).

The conclusions should be taken with cautions in light of several limitations. Above all, the G × E interaction effect detected in the present study is quite small, which is commonly demonstrated in gene–environment interaction studies. And no wonder since there may be many intermediate variables between genes and aggressive behavior, such as catecholamine availability, cognition, emotional control, and so on. Given the lower number of the *COMT* GT and TT genotypes in this study, these two genotypes were pooled. Therefore, it still remained unclear whether there were significant differences in the effects of heterozygous GT and homozygous TT on adolescent aggressive behavior. The findings of the present study are based on cross-sectional assessments, so longitudinal studies are needed in order to establish the direct causal link between SLEs and aggressive behaviors, as well as to examine the longitudinal effects of *MAOA* and *COMT* polymorphisms and SLEs on adolescents’ aggressive behaviors over time. The assessment of SLEs in this investigation was limited to the past 12 months. It will be meaningful to examine the interactional effects of genes and SLEs on aggressive behaviors using different measures, such as early SLEs occurring in childhood and all the SLEs occurring in the lives of participants. We didn’t have the specific measures at the level of class, teacher, or school (the nested structure data), it will be important in the future to examine whether and how the variables at non-individual levels influence the associations observed in the current study using multilevel linear models. It is also possible that *MAOA* and *COMT* genes are markers for haplotypes, and that genes in linkage disequilibrium with *MAOA* and *COMT* are responsible for the association with aggressive behavior. Nevertheless, the present study also had some strengths. Most notably, it conducts the research in a large population-based sample, and its reliability to detect the main and interaction effect was enhanced because of the correction for multiple testing. Furthermore, this study collects the aggressive behavior data from both adolescents’ mothers and their teachers, which decreases or avoids the risk of information bias.

## Conclusion

The current study provides the first evidence of *COMT* × *MAOA* × SLE interaction effect on male adolescents’ aggressive behaviors. Although future studies are needed both to replicate this effect and to clarify its possible biological pathway, our findings demonstrated that the *MAOA* T941G polymorphism interactive with SLEs does contribute to adolescents’ aggressive behaviors, and this interactive effect was moderated by *COMT* Ala22/72Ser polymorphism. This study also highlights the importance of considering distinct domains of stressful events and information bias when examining the effect of *MAOA* and *COMT* on aggressive behavior.

## Author Contributions

MW, HL, and WZ contributed to the conception and design of the study. MW performed the data analyses and drafted the manuscript. HL, KD-D, and WZ provided critical revisions. All authors approved the final version of the manuscript for submission.

## Conflict of Interest Statement

The authors declare that the research was conducted in the absence of any commercial or financial relationships that could be construed as a potential conflict of interest.
